# Systemic immunity shapes the oral microbiome and susceptibility to bisphosphonate-associated osteonecrosis of the jaw

**DOI:** 10.1186/s12967-015-0568-z

**Published:** 2015-07-04

**Authors:** Shirin Kalyan, Jun Wang, Elgar Susanne Quabius, Jörn Huck, Jörg Wiltfang, John F Baines, Dieter Kabelitz

**Affiliations:** 1 Institute of Immunology, Christian-Albrechts University Kiel, Arnold-Heller-Strasse 3, Bldg. 17, 24105 Kiel, Germany; 2 Max Planck Institute for Evolutionary Biology, August-Thienemann-Str. 2, 24306 Plön, Germany; 3 Institute for Experimental Medicine, Christian-Albrechts-University of Kiel, Arnold-Heller-Str. 3, Bldg. 17, 24105 Kiel, Germany; 4 Department of Otorhinolaryngology, Head and Neck Surgery, Christian-Albrechts University Kiel, Arnold-Heller-Str. 3, Bldg. 27, 24105 Kiel, Germany; 5 Department of Oral and Maxillofacial Surgery, Christian-Albrechts University Kiel, Arnold-Heller-Str. 3, Bldg. 26, 24105 Kiel, Germany

**Keywords:** Adverse drug effects, Bisphosphonates, Immune function, Oral microbiome, Osteonecrosis of the jaw

## Abstract

**Background:**

Osteonecrosis of the jaw (ONJ) is a rare but serious adverse drug effect linked to long-term and/or high-dose exposure to nitrogen-bisphosphonates (N-BP), the standard of care for the treatment of bone fragility disorders. The mechanism leading to bisphosphonate-associated ONJ (BAONJ) is unclear and optimal treatment strategies are lacking. Recent evidence suggests that BAONJ may be linked to drug-induced immune dysfunction, possibly associated with increased susceptibility to infections in the oral cavity. The objective of this investigation was to comprehensively assess the relationship linking immune function, N-BP exposure, the oral microbiome and ONJ susceptibility.

**Methods:**

Leukocyte gene expression of factors important for immunity, wound healing and barrier function were assessed by real-time quantitative PCR and the oral microbiome was characterized by 454 pyrosequencing of the 16S rRNA gene in 93 subjects stratified by N-BP exposure and a history of ONJ.

**Results:**

There were marked differences in the systemic expression of genes regulating immune and barrier functions including *RANK* (*p* = 0.007), aryl hydrocarbon receptor (*AHR*, *p* < 0.001), and *FGF9* (*p* < 0.001), which were collectively up-regulated in individuals exposed to N-BP without ONJ relative to treatment controls. In contrast, the expression levels of these same genes were significantly down-regulated in those who had experienced BAONJ. Surprisingly, the oral microbiome composition was not directly linked to either BAONJ or N-BP exposure, rather the systemic leukocyte expression levels of *RANK*, *TNFA* and *AHR* each explained 9% (*p* = 0.04), 12% (*p* = 0.01), and 7% (*p* = 0.03) of the oral bacterial beta diversity.

**Conclusions:**

The oral microbiome is unlikely causative of ONJ, rather individuals with BAONJ lacked immune resiliency which impaired their capacity to respond adequately to the immunological stress of N-BP treatment. This may be the common factor linking N-BP and anti-RANK agents to ONJ in at-risk individuals. Preventive and/or therapeutic strategies should target the wound healing deficits present in those with ONJ.

**Electronic supplementary material:**

The online version of this article (doi:10.1186/s12967-015-0568-z) contains supplementary material, which is available to authorized users.

## Background

Since their approval for clinical use in the mid-1980’s, nitrogen-bisphosphonates (N-BP) have become the established treatment for diseases of excess bone resorption such as osteoporosis, Paget’s disease and cancer-associated bone disease. N-BP are structural analogues to pyrophosphates, which confers their ability to bind to hydroxyapatite crystals of bone surfaces [1]. The potent anti-bone resorption property of N-BP is mediated through their inhibition of a key enzyme in the mevalonate pathway for isoprenoid biosynthesis, farnesyl pyrophosphate synthase (FPPS) [2, 3], which results in the prevention of protein prenylation necessary for osteoclast function [4].

Despite clinical efficacy for the prevention of certain types of fragility fractures [5, 6], high-dose or long-term N-BP exposure is associated with rare but serious drug-related complications such as osteonecrosis of the jaw (ONJ) [7, 8]. ONJ appears as a painful lesion of exposed necrotic bone in the mandible or maxilla that fails to heal within 8 weeks [9]. The exact mechanism leading to bisphosphonate-associated ONJ (BAONJ) is unknown, but one prevalent theory is that it may be induced by the direct toxic effects of the drug on bone and soft tissue [10]. However, emerging reports of ONJ in patients treated with the new anti-resorptive agent, anti-RANKL antibody (denosumab) [11]—a biologic with a different mechanism of action, raises doubts that BAONJ is primarily a consequence of drug-induced tissue toxicity. Proposed risk factors include periodontal disease, smoking, cancer, chemotherapy, glucocorticoid use and diabetes [12].

We previously noted that osteoporosis patients treated with N-BP tended to become deficient in a unique subset of human peripheral blood innate T cells bearing the Vγ9 Vδ2 TCR [13]. This loss was directly correlated to the length of time on therapy and was most striking in patients receiving the drug intravenously [13]. The observed decline of γδ T cells might be at least partially due to inhibitory effects of granulocytes on Vγ9 Vδ2 T cell activation upon up-take of N-BP [14, 15]. A suppressive effect of granulocytes on human γδ T cell activation has been recently observed by other groups as well [16]. Patients who had experienced ONJ were largely lacking peripheral Vγ9 Vδ2 T cells, but they all additionally had co-morbid conditions that could further impair immune resiliency [13]. In this study, we evaluated the impact of N-BP treatment and the occurrence of BAONJ on leukocyte mRNA expression patterns of genes that are pivotal for immunity, wound healing and/or barrier function known to be, in part, regulated by γδ T lymphocytes [17–24]. Collectively, these included *RANK*, *RANKL*, *TNFA*, *IL17*, *IFNG*, *IL1B*, *FGF9*, *GMCSF*, *CTGF*, *MMP7*, *MMP9*, and *AHR*.

The oral microbiota (and in consequence oral immunity) has been repeatedly postulated to be linked to the development of BAONJ [25–27], but there is no compelling evidence showing a direct relationship between infection and ONJ pathogenesis [28]. Nevertheless, aggressive systemic treatment with antibiotics is usually part of the therapeutic strategy to manage ONJ. This approach appears to have limited impact on the overall oral microbial diversity [29], but it potentially provides some prophylactic benefit for patients with multiple myeloma given N-BP intravenously [30].

The aim of our present study was to define the relationship linking immune and wound healing resiliency, N-BP exposure, the oral microbiome and susceptibility to BAONJ to (1) improve identification of those individuals at greatest risk for the development of ONJ, and (2) provide mechanistic insight into its pathophysiology to advance current treatment strategies.

## Methods

### Study design

This study was approved by the Clinical Ethics Board of the Faculty of Medicine of Christian-Albrechts University of Kiel (D 411/11) and was conducted in accordance with the principles of the Declaration of Helsinki for medical research involving human subjects. Patients with osteoporosis who were either N-BP-treatment naive or had been on continuous N-BP therapy for various lengths of time were invited to participate in the study through a network of community physicians in Kiel, Germany. The number of subjects in each treatment group was based on our previous findings on the observable differences in immune profiles [13]. The diagnosis and treatment of osteoporosis were based on the guidelines established by the German Specialist Organisation for Osteology (Dachverband Osteologie, DVO [31]. Exclusion criteria for the osteoporosis cohort included use of strong immuno-modulatory drugs such as systemic corticosteroids or other immunosuppressive agents, and the presence of any malignancy. Patients who had experienced N-BP-associated ONJ within the last 2 years were contacted through the Department of Oral and Maxillofacial Surgery. The diagnosis of BAONJ was based on established guidelines [8]. The only exclusion criterion for the BAONJ cohort was being on active antibiotic treatment. Additional control subjects who were age-matched for the BAONJ cohort were later recruited through physician offices and the Department of Immunology to account for the potential influence of age on the variables assessed. All study subjects provided informed written consent.

### Sample collection

Blood collected in EDTA tubes was aliquoted in 200 µl and mixed with 800 µl Prisure (Promolgene, Berlin, Germany) and immediately stored at −80°C for subsequent RNA extraction, cDNA synthesis and gene expression analysis. Isohelix DNA swabs (SK-2S, Biolab products GmbH, Bebensee, Germany) were used to obtain microbial DNA by running the swab along the same area of the outer gumline of subjects (original instructions provided to physicians for uniform oral microbiome sampling is provided as Additional file 1). DNA extraction was performed using the Isohelix Buccalyse DNA extraction kit (Biolab products GmbH, Bebensee, Germany). The extracted DNA was stored at −20°C until analysis.

### RNA extraction

RNA extraction was carried out on defrosted blood samples using the Prisure reagent (Promolgene, Berlin, Germany) following the manufacturer’s protocol. The resulting RNA pellet was dissolved in 20 µl diethylpyrocarbonate-(DEPC-) treated water (Promolgene, Berlin, Germany), and RNA concentration was measured using the Nanodrop 1000 (Peqlab, Erlangen Germany). The remaining RNA was stored at -20°C until cDNA synthesis.

### cDNA synthesis

For cDNA synthesis, 200 ng total RNA were transcribed into cDNA, using the TR cDNA synthesis kit (AmpTec Hamburg, Germany) with the provided oligo dT-V primer. cDNA synthesis was carried out using a Thermocycler I (Biometra, Göttingen, Germany) as follows: primer and RNA were incubated at 65°C for 5 min; samples were immediately placed on ice and the PCR mix (RT-enzyme, RT-buffer, and dNTPs) was added; lastly samples were incubated for 60 min at 37°C, followed by a denaturation step (10 min at 72°C). The resulting cDNAs were purified from the remaining enzyme, buffer, and dNTPs with the provided spin columns and stored at −20°C until further analysis.

### Real-time quantitative polymerase chain reaction (RTqPCR)

RTqPCR was performed using a Rotorgene 3000 (Corbett, LTF, Wasserburg, Germany). SYBR green-based qPCR mix and primers for the housekeeping genes (b-actin, beta-2-microglobulin and 18S) were purchased from Promolgene (Berlin, Germany). All PCR reactions were run in duplicates using 2.5 µl of the above mentioned cDNA (≈10 ng total RNA) in a total reaction volume of 25 µl. PCR conditions were as follows: 10 min initial denaturation at 95°C, followed by 40 cycles of denaturation: 20 s at 95°C, annealing 20 s at primer specific annealing temperatures (see below), elongation: 20 s at 72°C. After the last cycle, a melt curve analysis was performed starting at the primer specific annealing temperatures. Unless otherwise indicated, primers were designed using the web-based primer3 software (http://primer3.wi.mit.edu/) and were synthesized by TIB MOLBIOL (Berlin, Germany). The following primers for the respective genes (annealing temperatures provided in parenthesis) were assessed: *FGF9* sense primer: 5′-GGCGTGGACAGTGGACTCTACCTC -3′, *FGF9* antisense primer: 5′-TTCCCATCCAAGCCTCCATCATAC -3′ (56°C) [18]; *IFNG* sense primer: 5′-TCAGCTCTGCATCGTTTTGG-3, *IFNG* antisense primer: 5′-GTTCCATTATCCGCTACATCTGAA-3′ (60°C); *TNFA* sense primer: 5′-CTTCTCGAACCCCGAGTGA-3′, TNFα antisense primer: 5′-CCTCTGATGGCACCACCAG-3′ (60°C); *IL1B* sense primer: 5′-CTGTCCTGCGTGTTGAAAGA-3′, *IL1B* antisense primer: 5′-TTGGGTAATTTTTGGGATCTACA-3′ (62°C); *IL17* sense primer: 5′-TTAAGGCCCCTCAGAGATCA-3′, *IL17* antisense primer: 5′-TCAGCTCCTTTCTGGGTTGT-3′ (64°C); connective tissue growth factor (*CTGF*) sense primer: 5′-ACGGCGAGGTCATGAAGAAGAACA-3′, *CTGF* antisense primer: 5′-TGGGGCTACAGGCAGGTCAGTG-3′ (61°C) [19]; *RANKL* sense primer: 5′-ACCAGCATCAAAATCCCAAG3′, *RANKL* antisense primer: 5′-ATCCAGTAAGGAGGGGTTGG-3′ (62°C); *RANK* sense primer: 5′- AGGGAGCATGTGAAGGTGTC-3′, *RANK* anti sense primer 5′-TGCTGACCAATGAGAGCATC-3′ (64°C); *MMP7* sense primer: 5′-TCAGGCAGAACATCCATT-3′, *MMP7* antisense primer: 5′-TTTATTGACATCTACCCAACTGC-3′ (50°C) [20]; *MMP9* sense primer: 5′-CGCGGGCGGTGATT G ACGAC-3′, *MMP9* antisense primer: 5′-GTGGTGCAGGCGGAGTAGGATTGG-3′ (63°C) [20]; *GMCSF* sense primer: 5′-TGCTCTTGGGCACTGTGG-3′, *GMCSF* antisense primer: 5′- CCCTGCTTGTACAGCTCCAG-3′ (60°C) and aryl hydrocarbon receptor (*AHR*) sense primer: 5′-GTTGGACGTCAGCAAGTTCA-3′, *AHR* antisense primer: 5′-TGGTGCCCAGAATAATGTGA-3′ (60°C). Threshold levels for C_t_-determination were chosen manually. Data analysis was performed according to the ∆C_t_-method using the mean C_t_ value of three housekeeping genes [32].

### Microbiota sequencing and sequence filtering

The V1–V2 region of the bacterial 16S rRNA gene was amplified from the DNA extracted from mouth swabs using using primers 27F (*CTATGCGCCTTGCCAGCCCGC*TCAGTCAGAGTTTGATCCTGGCTCAG-3′) and 338R (5′*CGTATCGCCTCCCTCGCGCCA*TCAGXXXXXXXXXXCATGCTGCCTCCCGTAGGAGT-3′), where XXXX denotes a 10nt index sequences and the italicised sequences are adapters for the 454 Roche platform. Amplification was carried out using the Phusion Hot Start DNA Polymerase II (Finnzymes, Espoo, Finland) with the following PCR conditions: initial denaturation for 30 s at 98°C; 30 cycles of 9 s at 98°C, 30 s at 55°C, and 30 s at 72°C; final extension for 10 min at 72°C [33]. PCR products were then processed on a 454-FLX sequencer and output sequences were filtered according to quality (average >25) and length (minimum length 250, maximum length 400) using Mothur [34]. We obtained an average of 2,436 high quality reads per sample (minimum number was 955). To standardize the sample size, a subset of ~1000 reads was selected for each sample for the oral microbiota analysis, as suggested by Hamady et al. [35]. Chimera detection was performed using Uchime [36] against recommended databases. Classification of the sequences was carried out using RDP classifier [37] and taxonomical consensus was created for all samples from the phylum to genus level.

### Ecological analysis

Bray-Curtis and Jaccard dissimilarities were used to analyze beta diversity, and tests for differences among groups of samples were performed using constrained analysis of principle coordinates [38] (function “capscale”) and analysis of dissimilarity (function “adonis”) in the ‘VEGAN’ R package, R Development Core Team 2011 [39].

### Statistical analysis

Calculations for the fold change in gene expression assessed by RTqPCR used for graphical representation of the data were based on the mathematical model previously described [40]. Normalized ∆C_t_ values for each gene were checked for skewness and kurtosis. *RANK*, *RANKL*, *CTGF*, *MMP7*, *MMP9* and *AHR* showed a right skewed distribution and were log10 transformed for subsequent statistical analysis. Group differences in relative gene expression were assessed by ANOVA. Tukey’s HSD post hoc test was used to determine individual mean differences between cohorts while correcting for multiple comparisons. Pearson’s correlation was used to assess associations among variables, and multiple linear regression models were built for genes showing strong correlations with length of time on N-BP therapy with age included in the model. Generalized linear models were built to assess the relationship between leukocyte gene expression and bacterial taxa, while the Vegan software “envfit” function was used to assess the influence of the of leukocyte gene expression levels on oral bacterial communities. Data analysis was performed using SPSS version 20 (SPSS Inc., Chicago, IL, USA), and bacterial community comparisons were carried out using the “Vegan” R package (R Development Core Team 2011) [39].

## Results

### Study subjects

The study included 93 subjects stratified by exposure to N-BP and the occurrence of BAONJ within the last 2 years; detailed characteristics of study participants are provided in Table 1. Seventy-five of the 79 subjects (95%) with osteoporosis were postmenopausal women (plus 4 men with age-related osteoporosis), as were the 6 individuals who had previously been diagnosed with BAONJ and the 8 additional controls included for age-matching the slightly younger members of the BAONJ cohort. This reduced variance due to ovarian hormones on the immune and wound healing factors investigated. The subjects who had experienced BAONJ were sampled a median of 375 (range 1–606) days after diagnosis and treatment for BAONJ, and the majority continued N-BP treatment after BAONJ diagnosis.

**Table 1:** Characteristics of study subjects (n = 93) stratified by N-BP exposure and the occurrence of bisphosphonate-associated osteonecrosis of the jaw (ONJ)

Group	N	Mean age (years ± SD)	Median length of time on N-BP therapy (days, range)	Characteristics and type of N-BP therapy
N-BP treatment naïve controls	26	66.4 ± 10.0	NA	18 with osteoporosis (mean age 72.1 ± 6)
				8 Postmenopausal women without osteoporosis (mean age 53.6 ± 2)
Oral N-BP	30	74.1 ± 5.7	90 (1,764)	90% On oral alendronate (70 mg/week); 10% on oral ibandronate (150 mg/months)
Intravenous N-BP	31	73.2 ± 5.5	813 (1,982)	All on intravenous ibandronate (3 mg/months)
ONJ	6	61.3 ± 12.5	1,113 (1,282)	1 Woman (85 yrs) was being treated with 5 mg/year intravenous zoledronate (had received 2 shots) for osteoporosis, previously had a blood malignancy and was on prednisone
				1 Woman on systemic corticosteroids and mycophenolate mofetil on oral aledronate (70 mg/week) for 3.5 years
				1 Woman on interferon therapy for hepatitis B & C on oral aledronate (70 mg/week) for 5 years
				3 Women who had been treated for metastatic breast cancer; all on 4 mg/months intravenous zoledronate (from 1.4 to 2.8 years)

### Leukocyte gene expression of factors important for immunity, wound healing and barrier function

**Figure 1:**
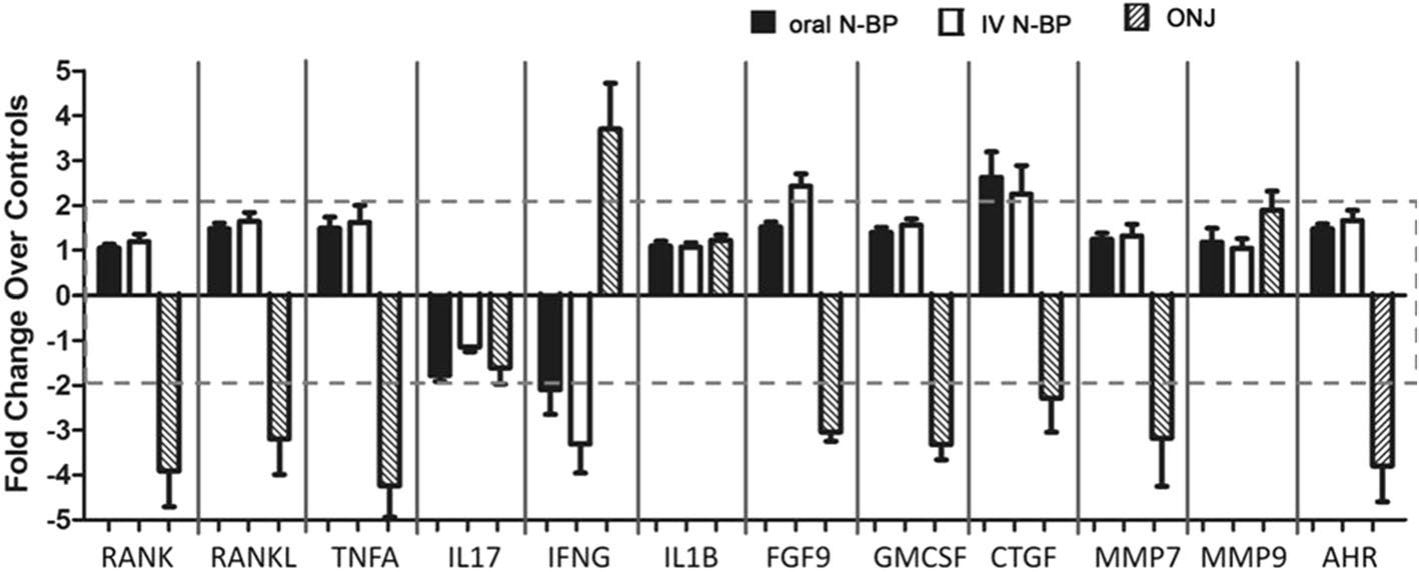
Relative leukocyte expression of genes important for immunity, wound healing and barrier function in subjects grouped by type of N-BP exposure (n = 30 oral, n = 31 intravenous) and recent history of osteonecrosis of the jaw (ONJ, n = 6). Data presented are the fold change in gene expression relative to the N-BP-treatment naive controls (n = 26). Significant overall group differences were observed for *RANK* (*p* = 0.007), *RANKL* (*p* = 0.026), *TNFA* (*p* = 0.001), *FGF9* (*p* < 0.001), *GMCSF* (*p* = 0.001), *CTGF* (*p* = 0.013), *MMP7*, and *AHR* (*p* < 0.001). The greatest mean difference in each case was between the intravenously N-BP treated subjects and those who had experienced ONJ with the latter consistently having the lowest levels of these genes relative to others. Only for the mRNA levels of *MMP7* was the main difference only between the intravenous N-BP and the ONJ cohorts as the expression levels of oral N-BP subjects and treatment controls did not differ statistically in comparison to any other group. The actual ΔC_T_ values are presented in the Table 2.

N-BP exposure has a marked impact on the immune system largely through their ability to activate a unique subset of human peripheral blood γδ T cells that bear the Vγ9 Vδ2 TCR [13, 41]. Previously it was shown the main consistent difference in the immune profile of those who had developed BAONJ was the almost complete loss of these cells in circulation, presumably through chronic or high-dose exposure to N-BP [13]. We therefore evaluated the impact of N-BP treatment and the occurrence of BAONJ on leukocyte mRNA expression patterns of genes that are pivotal for immunity, wound healing and/or barrier function (Figure 1) [17–24]. All individuals who had experienced BAONJ were conspicuously deficient in the expression of a specific subset of these factors—which included receptor activator of nuclear factor-κB (*RANK*), *RANK*-ligand (*RANKL*), tumor necrosis factor-alpha (*TNFA*), fibroblast growth factor-9 (*FGF9*), granulocyte–macrophage colony stimulatory factor (*GMCSF*), connective tissue growth factor (*CTGF*), matrix metalloproteinase-7 (*MMP7*), and the aryl hydrocarbon receptor (*AHR*); Figure 1 and Table 2. In contrast, individuals on N-BP treatment without a history of ONJ tended to up-regulate the expression of these same genes relative to treatment naive controls, and this effect was greatest in those on intravenous N-BP. Multiple linear regression analysis performed only on the osteoporosis cohort without a history of ONJ (n = 79) indicated that the length of time on N-BP therapy, but not age, was significantly linked to both higher *RANK* (*β* = 0.233, *p* = 0.045) and *AHR* (*β* = 0.247, *p* = 0.032) gene expression.

**Table 2:** ΔC_t_ values of the systemic expression of genes important for immunity, wound healing and barrier function in subjects (N = 93) stratified by exposure to N-BP and the occurrence of N-BP-associated osteonecrosis of the jaw (ONJ)

	Controls (n = 26)	Oral N-BP (n = 30)	i.v. N-BP* (n = 31)	ONJ (n = 6)	*p* value
Cytokines					
*RANK* ^a^	0.95 ± 0.09	0.95 ± 0.03	0.94 ± 0.06	1.0 ± 0.13*	0.026
*RANKL* ^a^	1.0 ± 0.08	0.98 ± 0.04^	0.97 ± 0.05^^	1.1 ± 0.1	0.007
*TNFA*	8.1 ± 1.4	7.6 ± 1.3	7.5 ± 1.8	10.2 ± 1.7	0.001
*IL17*	12.6 ± 1.6	13.4 ± 0.97	12.8 ± 1.1	13.3 ± 3.0	0.129
*IFNG*	13.8 ± 4.5	14.8 ± 3.9	15.5 ± 3.0	11.9 ± 1.3	0.109
*IL1B*	10.8 ± 1.4	10.7 ± 1.1	10.7 ± 0.96	10.5 ± 1.1	0.942
Growth and wound healing factors					
*FGF9*	11.3 ± 1.8	10.7 ± 0.8	10.1 ± 1.1***	12.9 ± 0.9*	<0.001
*GMCSF*	14.7 ± 1.6	14.2 ± 1.1	14.0 ± 1.3	16.4 ± 1.7*	0.001
*CTGF* ^a^	0.86 ± 0.14	0.79 ± 0.10	0.80 ± 0.11	0.93 ± 0.14	0.013
*MMP7* ^a^	0.86 ± 0.08	0.85 ± 0.05	0.84 ± 0.08^	0.93 ± 0.16	0.040
*MMP9* ^a^	0.96 ± 0.07	0.95 ± 0.10	0.96 ± 0.09	0.92 ± 0.10	0.611
Response to challenge (endogenous or exogenous)					
*AHR* ^2^	0.93 ± 0.06	0.90 ± 0.03	0.89 ± 0.05*	1.0 ± 0.10**	<0.001

ΔC_t_ values decrease with increased gene expression; lower values correspond to greater gene expression and higher values to lower gene expression.

Tukey HSD post hoc test: * significantly different from controls, *p* < 0.05; ** significantly different from controls, *p* < 0.01; *** significantly different from controls, *p* < 0.005; ^ significantly different from ONJ, *p* < 0.05; ^^ significantly different from ONJ, *p* < 0.01. The differences between the N-BP treatment groups and ONJ are only shown for factors where there was no significant difference with the control group. The greatest differences were observed between N-BP treated groups without a history of ONJ and those with ONJ.

*i.v.* intravenous.

^a^Data are log10 transformed.

**Figure 2:**
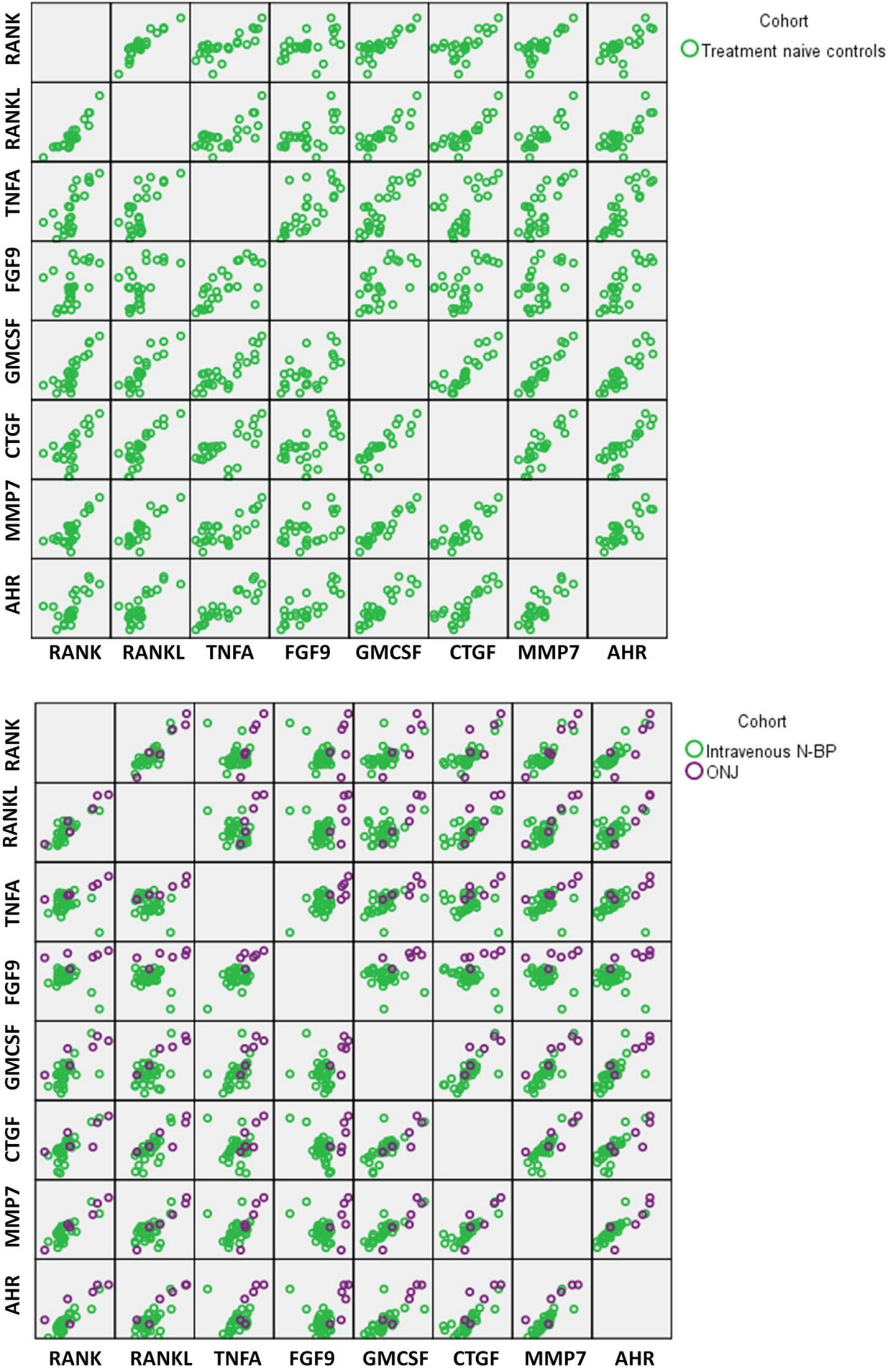
Scatter plot matrix showing the relationship amongst genes found to be differentially expressed in ONJ subjects. *Top panel* N-BP treatment naive controls show large inter-individual variance in the leukocyte expression levels of the immune and wound healing genes, which show a high degree of correlation. *Bottom panel* intravenous N-BP exposure (*green*) perceptibly reduced within-group variance in expression levels which were shifted to being highly clustered; ONJ cases (*purple*), however, clearly failed to respond similarly to N-BP exposure and the immune and wound healing gene expression levels remained markedly reduced. Note- data represent the actual normalized C_t_ values, thus higher values moving *left* to *right* within the matrix correspond to lower gene expression. Statistical differences between groups are reported in Table 2.

Visualizing the mRNA expression of these differentially regulated genes on a scatter plot matrix shows that most are highly correlated, and in the treatment control subjects, the inter-individual expression pattern is relatively diverse (Figure 2, top panel). In contrast, exposure to N-BP, especially intravenous treatment, noticeably reduced this inter-individual variance and shifted the gene expression to a highly clustered pattern for each factor (Figure 2, bottom panel). However, those who had experienced BAONJ were clearly differentiated as a separate subset who failed to respond similarly to N-BP treatment (Figure 2, bottom panel).

### Composition of the oral microbiota in relation to N-BP exposure and the development of BAONJ

**Figure 3:**
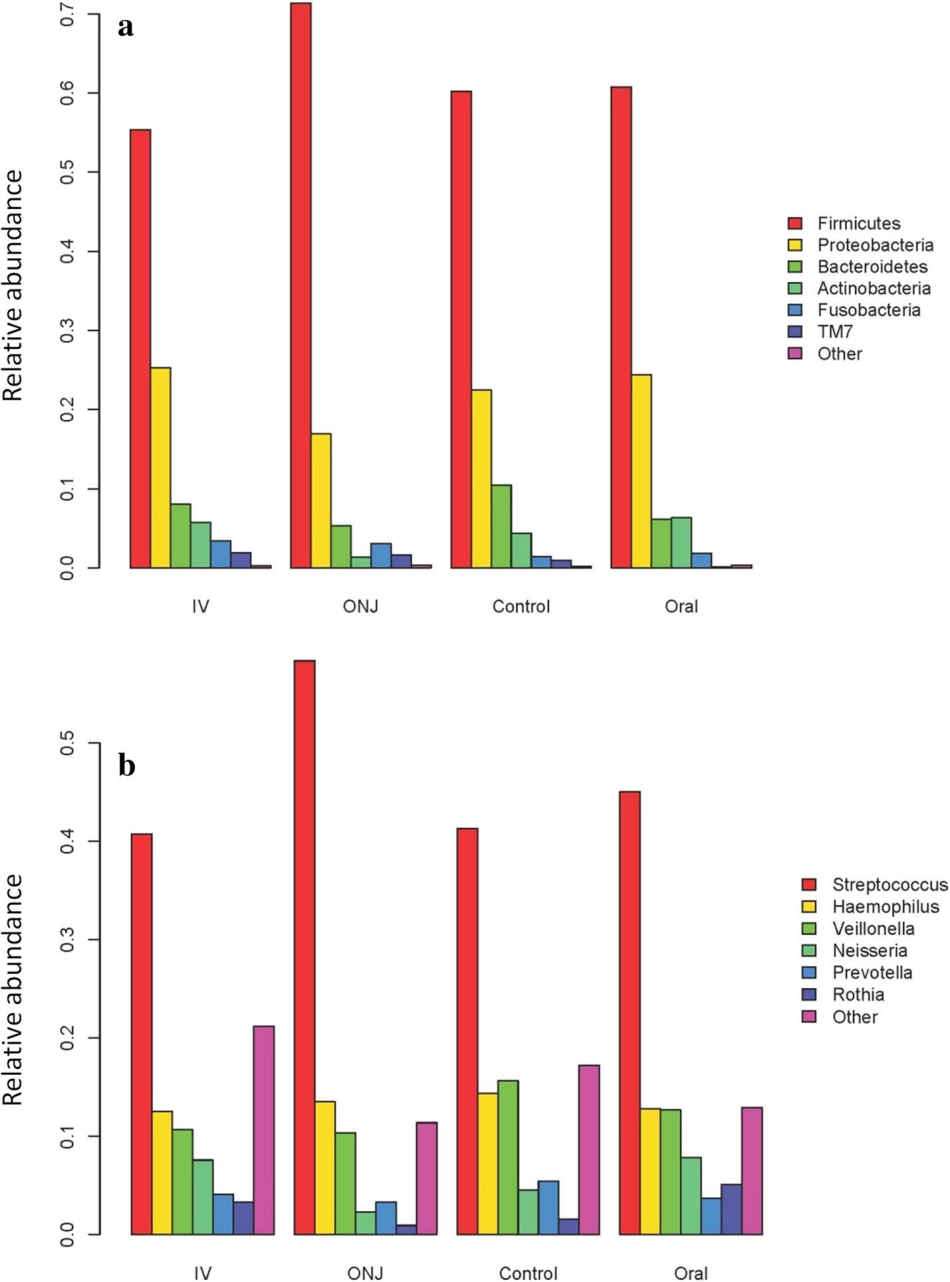
Oral microbiota profile of individuals stratified by N-BP exposure and history of N-BP-associated osteonecrosis of the jaw (ONJ). **a** At the phylum level, Firmicutes (59.02%) and Proteobacteria (24.27%) were the most prominent oral bacterial communities, followed by Bacteroidetes (7.82%), Actinobacteria (5.21%), Fusobacteria (2.35%) and TM7 (1.04%), an uncultured candidate division. The remaining phyla were <1% of the composite communities. **b** At the level of genera, *Streptococcus* (43.11%), *Haemophilus* (13.29%), *Veillonella* (12.68%), *Neisseria* (6.49%), *Prevotella* (4.28%), *Rothia* (3.21%) and unclassified Prevotellaceae (2.55%) were the most abundant genera, with the remainder having <2.5% average abundance. Variations in the composition of the oral microbiome between groups were not statistically different.

**Figure 4:**
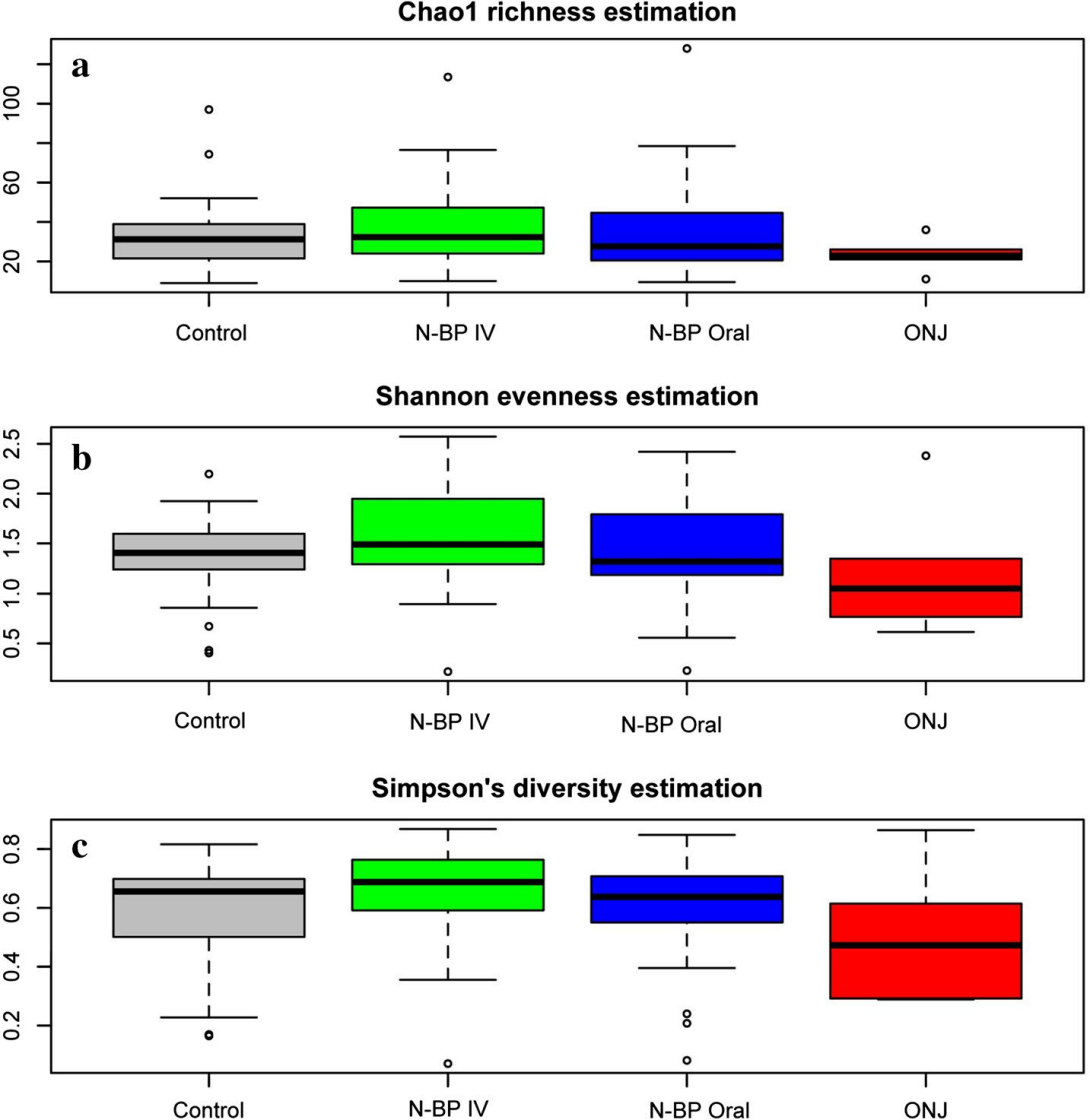
Alpha diversity measures of the plaque microbiome. Chao1 richness estimations (**a**), Shannon evenness (**b**) and Simpson’s diversity measures (**c**) are shown for each group. No significant differences can be found among all groups, nor ONJ group vs Control group, N-BP oral and N-BP IV group.

The composition of the oral microbiome was assessed by 454 pyrosequencing of the bacterial 16S rRNA gene, and a subset of 1,000 reads was selected for each sample for the analysis to standardize sample size [35]. The microbiome composition at phylum and genus level is shown in Figure 3 for each cohort. Subjects who had experienced BAONJ had the highest mean relative abundance of Firmicutes (71.32 versus 55.31–60.70%), which paralleled their higher abundance of *Streptococcus* (the major member of Firmicutes in the oral microbiome). Consequently, those who had experienced BAONJ also had lower Proteobacteria (16.93 versus 22.42–25.21%). However, these differences were not statistically significant and no obvious differences in other phyla were detected. These findings were similar when the analysis was performed with age-matching, with the exception the higher *Streptococcus* abundance was more pronounced in the BAONJ group relative to controls. For alpha diversities (microbiome composition richness and diversity), the BAONJ cohort had the lowest averages based on genus-level composition, including the richness estimator Chao1, evenness estimator, Shannon, as well as diversity estimator Simpson’s index (Figure 4).

In the context of bacterial communities, *beta diversity* refers to the extent to which taxa differ between groups. These differences may be either quantitative—taking abundances of each taxa into account (Bray-Curtis dissimilarity), or qualitative and based on the presence/absence of a given group (Jaccard dissimilarity). Using analysis of dissimilarity (function “adonis”) [39] that determines if intra-group dissimilarity are significantly lower than that of inter-group, we estimated whether oral bacterial communities differ among control groups, ONJ patients and patients with different N-BP exposures. In summary, neither of the dissimilarity measures of beta diversity showed any significant differences on the overall composition of bacterial communities based on stratification by N-BP exposure and a history of BAONJ (Bray-Curtis dissimilarity: adonis r = 0.021, p = 0.856; Jaccard dissimilarity: adonis r = 0.020, p = 0.936); and no significance was found even when comparing oral microbiota from age-matched controls and BAONJ patients (Bray-Curtis dissimilarity: adonis r = 0.043, p = 0.718; Jaccard dissimilarity: adonis r = 0.050, p = 0.694).

### The relationship between the oral microbiome and leukocyte gene expression

**Figure 5:**
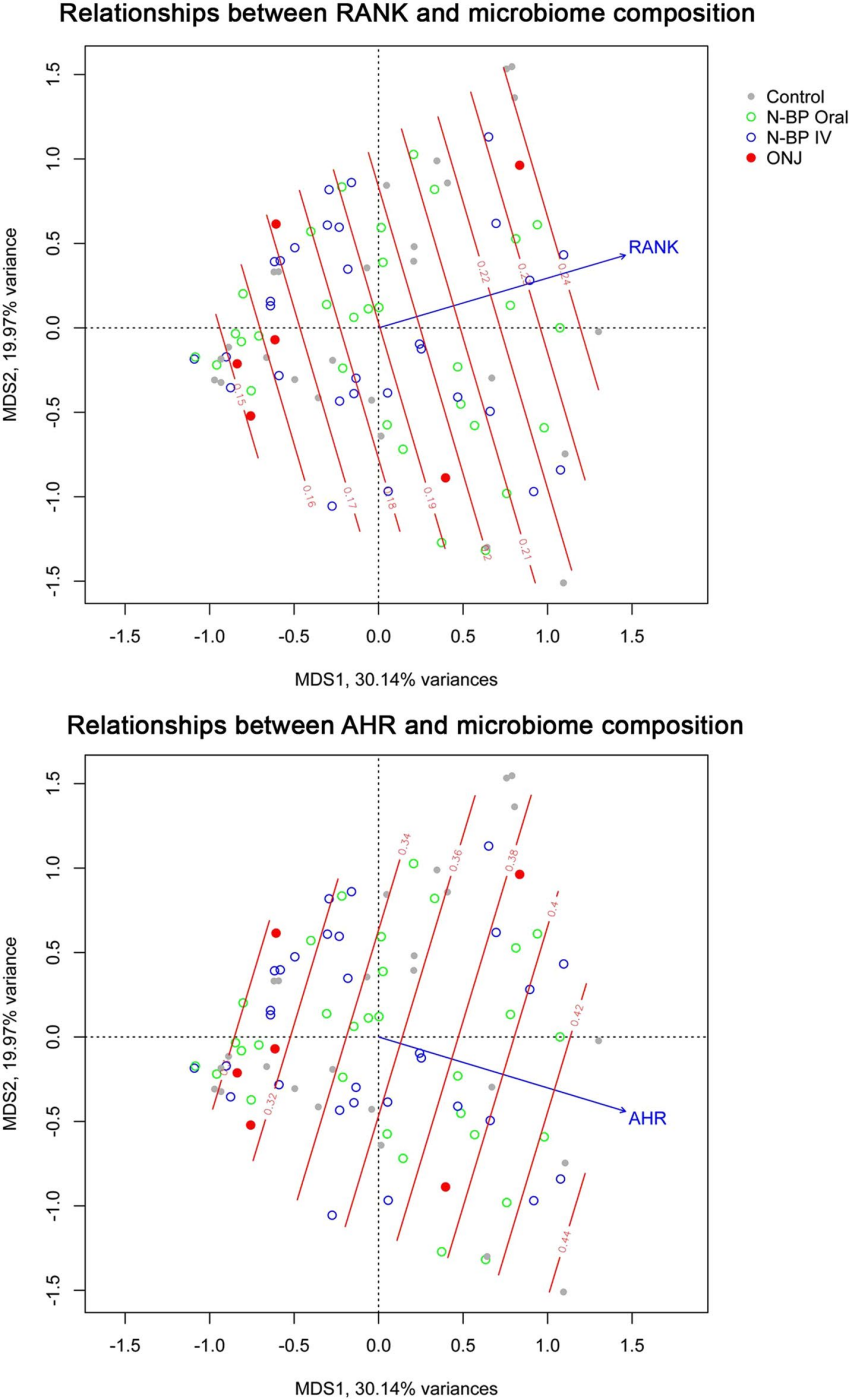
Systemic gene expression levels of *RANK* and *AHR* explain part of the variation observed in the oral microbiome. Ordinations of all microbial communities are plotted using principal coordinates analysis (*capscale*) based on Bray-Curtis dissimilarities. The contour of *RANK* (*upper panel*) and *AHR* (*lower panel*) gene expression levels are average values calculated using a multi-dimensional linear model in *odisurf,* and *arrows* indicate the direction of increasing gene expression.

The composition of the oral microbiota was observed to be strongly influenced by the peripheral blood leukocyte expression levels of a specific subset of immune and stress resiliency genes. *RANK*, *TNFA* and *AHR* each explained 9% (*p* = 0.04), 12% (*p* = 0.01), and 7% (*p* = 0.03) of the variance observed in the quantitative abundance of the bacterial groups. *RANK* and *AHR* expression also significantly contributed to qualitative differences of the oral microbiome across individuals. Figure 5 and Table 3 provides the Bray-Curtis and Jaccard dissimilarity *r* ^*2*^ values corresponding to the relationship between the gene expression levels and the composition of the oral microbiome. The abundance of *Streptococcus* was inversely related to *RANK* (r^2^ = 0.13, *p* = 0.0002) and *AHR* (r^2^ = 0.010, *p* = 0.0011) expression. In contrast, the abundance of *Fusobacterium* was positively associated to leukocyte *RANK* expression (r^2^ = 0.044, *p* = 0.024).

**Table 3:** The relationship between quantitative (Bray-Curtis Dissimilarity) and qualitative (Jaccard Dissimilarity) beta diversity measures of the oral microbiome and the expression of genes related to immune system, wound healing and barrier function

	*r* ^*2*^ for Bray-Curtis	*p* value	*r* ^*2*^ for Jaccard	*p* value
Cytokines				
*RANK*	0.0872	0.040	0.0723	0.028
*RANKL*	0.0611	0.089	0.0395	0.171
*TNFA*	0.1169	0.010	0.0176	0.542
*IL17*	0.0073	0.810	0.0138	0.573
*IFNG*	0.0256	0.419	0.0305	0.366
*IL1B*	0.0677	0.095	0.0364	0.192
Growth and wound healing factors				
*FGF9*	0.0271	0.281	0.0298	0.247
*GMCSF*	0.0391	0.168	0.0438	0.140
*CTGF*	0.0319	0.230	0.0404	0.144
*MMP7*	0.0235	0.328	0.0267	0.263
*MMP9*	0.0177	0.462	0.0245	0.298
Response to challenge (endogenous or exogenous)				
*AHR*	0.0721	0.027	0.0768	0.034

## Discussion

Serious adverse drug reactions often take several years to come to light after formal approval for clinical use [12]. The first published reports on BAONJ came out in 2003 [42]—more than a decade after the widespread clinical application of N-BP for a broad range of bone fragility disorders. The pathophysiological mechanism leading to BAONJ has been elusive, which has prevented the ability to clearly identify those specifically at risk and the implementation of effective preventative strategies. ONJ remains a significant challenge to treat and is usually dealt with surgically with a short-course of antibiotics. In our experience, multiple surgical attempts may be required with 40–50% of cases failing to heal or suffering a reoccurrence, which is similar to other published reports on treatment outcomes [43].

In this investigation, we show the unifying characteristic of having had a recent history of BAONJ was a systemic deficit in the expression of genes that together play a role in enforcing immunity, wound healing and barrier function. These genes are normally regulated in part by human peripheral blood γδ T cells, which are adversely affected in those with BAONJ [13]. Prominently included in this group were *AHR*, TNF family members (*RANK*, *RANKL* and *TNFA*) and mediators of wound and tissue healing (*FGF9*, *GMCSF*, *CTGF*, and *MMP7*). This strongly contrasted with our observation that N-BP exposure normally increased the mean expression levels of these same factors and reduced inter-individual variation in subjects with osteoporosis. In the latter group, the length of time on N-BP therapy, but not age, was significantly linked to increased leukocyte *RANK* and *AHR* expression, which is congruent with the notion that N-BP exposure exerts a stress on the immune system [44]. This finding may also explain the occurrence of ONJ in patients treated with anti-RANKL antibody [11], which similarly taxes the same immune-bone axis. RANK, often expressed on cells of monocytic lineage and osteoclasts, and its receptor, RANKL (also known as TRANCE; TNF-related activation induced cytokine), expressed on activated T cells and osteoblasts, play a central role in both bone remodeling and immune homeostasis, and the respective signaling cascade leads to activation of the NF-κB pathway [45, 46]. AHR, a transcription factor pivotal for xenobiotic metabolism, also cooperates in signaling through NF-κB to promote dendritic cell maturation [47], underscoring the highly integrated systems governing stress adaptation and immune regulation. AHR activity is an essential requirement for intraepithelial lymphocytes of the gut, particularly γδ T cells, and its deficiency resulted in the loss of control over microbial load and composition and epithelial cell turnover [22]. Furthermore, AHR can promote the expression of *FGF9* [48], which was one of the most conspicuously deficient factors in those who had experienced BAONJ. FGF9 is a growth factor important for tissue repair that plays a prominent role in the development of the neural crest-derived frontal bones of the skull [49], which makes it a promising therapeutic target to promote healing in those experiencing BAONJ.

Of note, the oral microbiome was not directly related to either N-BP exposure or ONJ status—but was instead associated with the gene expression levels of TNF family members and *AHR*. Investigations using a comprehensive high resolution metagenomic approach with appropriate control groups to assess the role of infection in BAONJ have been lacking. A study by Wei et al. utilizing a semi-quantitative technique based on gene fragmentation analysis on a denaturing gradient gel to compare the bacterial composition of the jawbone of 12 cancer patients (6 with BAONJ and 6 without ONJ or exposure to N-BP) found, similar to our results, that BAONJ was associated with a higher abundance of *Streptococcus* and lower *Fusobacterium*—with no other significant differences between the two groups [50]. More recently, Pushalkar et al., who similarly used denaturing gel analysis, observed that the oral microbiome diversity was lower in those who developed BAONJ (n = 5) compared to those with periodontal disease (n = 5) or those with a history of N-BP use without ONJ (n = 5) [51]. However, in addition to having a very small number of controls, this study failed to match for either sex or gender, which are important variables influencing both the microbiome and immune function. Similar to Wei et al’s study, we observed there was a greater abundance of *Streptococcus* but this change in microbiome composition was related to a reduced expression of genes important for barrier function, particularly *RANK*, *TNFA* and *AHR*. In contrast, *Fusobacterium*, a gram-negative anaerobe and a known contributor to periodontal disease, appears to thrive under conditions with higher expression of *RANK* and *AHR*, which may relate to the parallel increase in hypoxia with inflammation [52]. This provides clinical evidence that the oral microbiome is influenced by the systemic expression levels of these genes, and it supports a role for *AHR* in maintaining oral barrier function in humans, as was found to be the case for the murine gut [22]. This has important implications for the oral health of those who use pharmaceuticals or have disorders that exert a chronic pressure on systemic immunity.

BAONJ is a rare adverse drug effect of yet undetermined etiology; therefore the number of patients during the course of this investigation was limited. However, despite the heterogeneous medical history and demographic background of the ONJ patients included in the study, immune impairment (be it inherent or secondary to medication) was the primary consistent characteristic of its occurrence.

## Conclusions

Individuals who experienced BAONJ collectively lacked immune resiliency and normal barrier function as evidenced by their compromised gene expression levels for a number of pivotal factors—including *RANK*, *AHR* and *FGF9*. This reduced capacity to respond to challenge would not only affect their interaction with the normal microbiome, but it would also reduce their ability to withstand the significant added stress of N-BP treatment, which induced the up-regulation of these same stress response genes in those with normal immune function. This may be the common link between N-BP and anti-RANK agents and the development of ONJ in at-risk individuals. Intriguingly, the oral microbiome composition was strongly associated with the gene expression levels of *RANK* and *AHR* in leukocytes, but appears to be only indirectly linked to BAONJ and N-BP exposure. These results suggest those with either acquired (i.e., through drugs or illness) or inherent immune dysfunction are most at risk for the development of BAONJ and caution should be used when exposing such subjects to high-dose or long-term N-BP therapy. Preventative and treatment strategies should focus on targeting the immune and wound healing deficits observed in these patients.

### Additional file

**Additional file 1.** Instructions provided for the collection of the oral microbiome. For uniform collection procedures, physicians were provided a diagram illustrating where the mouth swab should sample along the gumline and how to store the samples for shipping back to the lab.

## Abbreviations

BAONJ: bisphosphonate-associated osteonecrosis of the jaw
N-BP: nitrogen-containing bisphosphonate(s)
ONJ: osteonecrosis of the jaw
