# Supplementary figures and images for: Systemic immunity shapes the oral microbiome and susceptibility to bisphosphonate-associated osteonecrosis of the jaw
Source: J Transl Med. 2015 Jul 4;13:212. doi: 10.1186/s12967-015-0568-z (PMC4490596; doi:10.1186/s12967-015-0568-z)

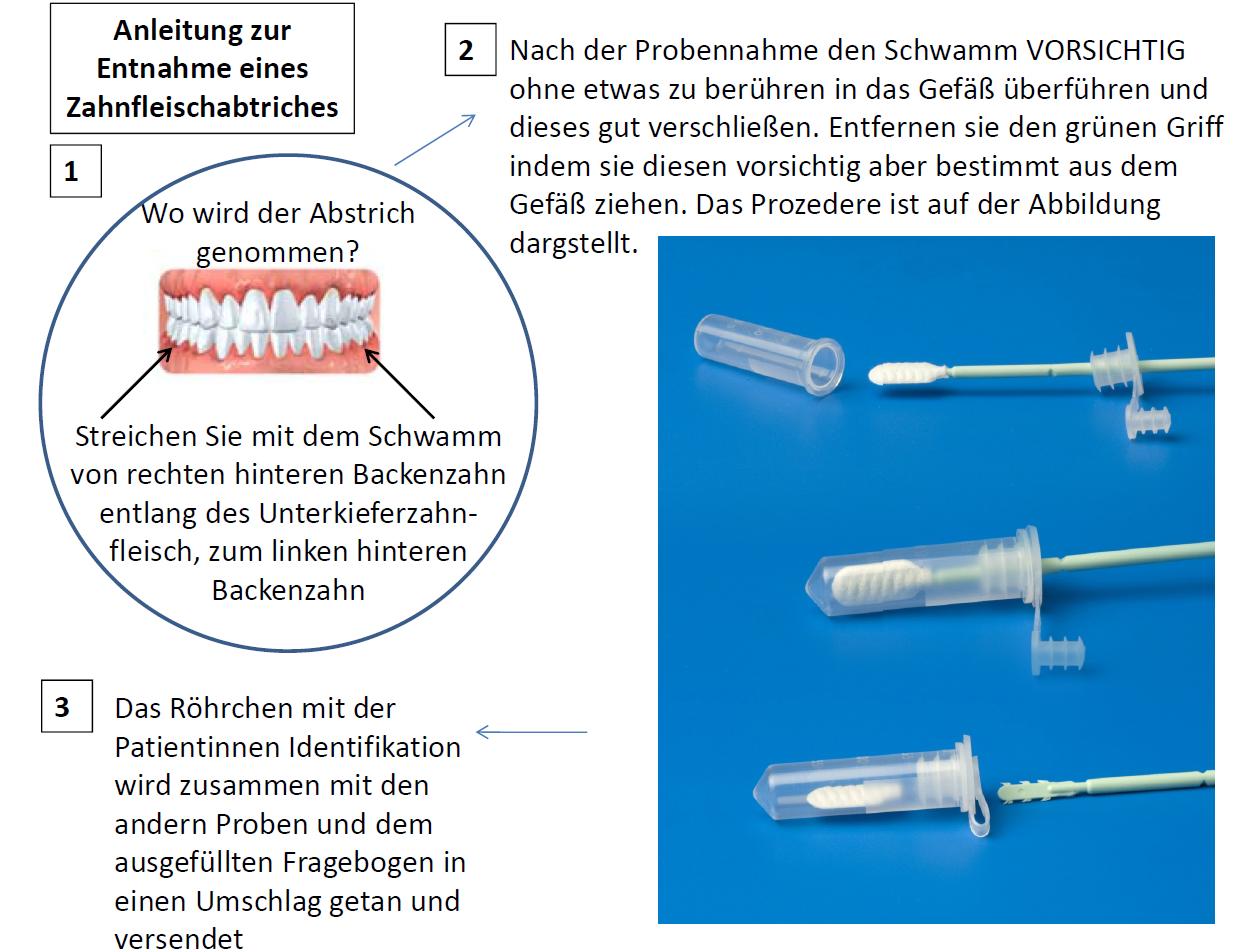

Supplement: Supplementary file 1 — Additional file 1. Instructions provided for the collection of the oral microbiome. For uniform collection procedures, physicians were provided a diagram illustrating where the mouth swab should sample along the gumline and how to store the samples for shipping back to the lab. [file 12967_2015_568_MOESM1_ESM.jpg]
